# Pathological Complete Response after Robot-Assisted Pulmonary Resection Following CDK4/6 Inhibitor–Combined Endocrine Therapy for Endobronchial Oligometastatic Breast Cancer: A Case Report

**DOI:** 10.70352/scrj.cr.26-0010

**Published:** 2026-03-26

**Authors:** Hikari Nitahara, Wataru Goto, Mariko Nishikawa, Chika Watanabe, Koji Takada, Yukie Tauchi, Kana Ogisawa, Haruhito Kinoshita, Tamami Morisaki, Kenichi Kohashi, Shinichiro Kashiwagi

**Affiliations:** 1 Department of Breast Surgical Oncology, Osaka Metropolitan University Graduate School of Medicine, Osaka, Osaka, Japan; 2 Department of Breast Surgery, Sumitomo Hospital, Osaka, Osaka, Japan; 3 Department of Pathology, Osaka Metropolitan University Graduate School of Medicine, Osaka, Osaka, Japan

**Keywords:** endobronchial metastasis, breast cancer, CDK4/6 inhibitor, pathological complete response, pulmonary resection

## Abstract

**INTRODUCTION:**

Endobronchial metastasis from breast cancer is rare. Even in oligometastatic disease, systemic therapy remains the standard treatment, and the role of surgical resection is not well established. We report a case of endobronchial oligometastatic breast cancer that achieved pathological complete response (pCR) after endocrine therapy combined with a cyclin-dependent kinase 4/6 (CDK4/6) inhibitor, followed by pulmonary resection.

**CASE PRESENTATION:**

A 48-year-old female with bilateral hormone receptor–positive, human epidermal growth factor receptor 2–negative breast cancer underwent surgery, followed by adjuvant chemotherapy and endocrine therapy. Two years and 8 months later, an elevated NCC-ST-439 level prompted further evaluation, which revealed a lesion in the right bronchus. Bronchoscopic biopsy confirmed metastatic breast cancer. No other metastatic lesions were detected, and the patient was diagnosed with endobronchial oligometastatic disease. Combination therapy with endocrine therapy and a CDK4/6 inhibitor resulted in tumor regression without new metastases. After 9 months of systemic therapy, robot-assisted right lower lobectomy with lymph node dissection was performed. Pathological examination revealed pCR. The patient remains progression-free 1 year and 9 months after surgery.

**CONCLUSIONS:**

This case suggests that surgical resection following effective systemic therapy may be a treatment option in selected patients with endobronchial oligometastatic breast cancer.

## Abbreviations

CDK4/6
: cyclin-dependent kinase 4/6
CR
: complete response
EBM
: endobronchial metastasis
ER
: estrogen receptor
HER2
: human epidermal growth factor receptor 2
HR
: hormone receptor
LH-RH
: luteinizing hormone–releasing hormone
MBC
: metastatic breast cancer
NG
: nuclear grade
pCR
: pathological complete response
PgR
: progesterone receptor
SUV
: standardized uptake value

## INTRODUCTION

The majority of metastatic lung tumors are parenchymal, whereas EBMs account for only approximately 5% of cases.^1)^ Colorectal and breast cancers are the most common primary tumors associated with pulmonary metastases, followed by uterine and renal cancers. Among patients with breast cancer, EBMs are particularly rare, occurring in only 0.4%–1.0% of cases.^2)^ Such lesions are typically observed in the terminal stages as part of widespread systemic dissemination, and oligometastatic presentations are uncommon.

Currently, MBC is considered an incurable disease, and systemic therapy remains the standard treatment approach.^3)^ Nevertheless, surgical resection of metastatic lesions in selected cases of oligometastatic disease has recently been discussed as a potential strategy to improve prognosis; however, supporting evidence remains limited.^4)^

Here, we report a rare case of endobronchial oligometastatic breast cancer in which pCR was achieved following endocrine therapy combined with a CDK4/6 inhibitor, after which pulmonary resection was performed.

## CASE PRESENTATION

A 48-year–old female underwent surgery for bilateral HR–positive, HER2–negative breast cancer at another institution (left breast: pT2N1M0, pStage IIB, NG 1, ly1, v0, ER/PgR/HER2 = 3b/3b/0, Ki-67 = 40%; right breast: pT1cN0M0, pStage IA, NG1, ly0, v0, ER/PgR/HER2 = 3b/3b/0, Ki-67 = 68%). She subsequently received adjuvant chemotherapy with docetaxel and cyclophosphamide for three months, followed by adjuvant endocrine therapy with tamoxifen.

**Fig. 1:**
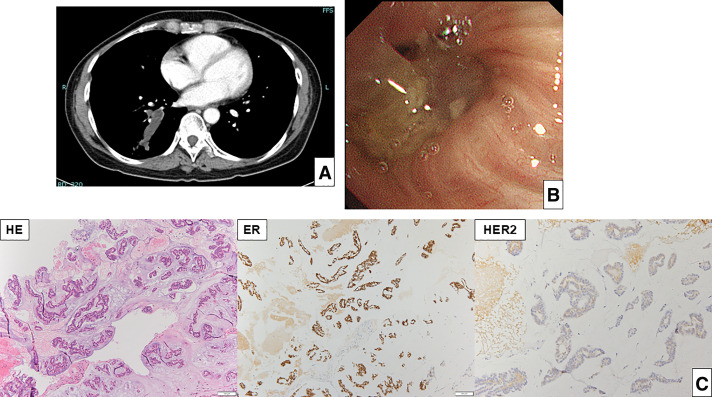
Pretreatment imaging findings. (**A**) Chest CT showing a lesion with mucin accumulation in the right bronchus (B10). (**B**) Bronchoscopic examination revealing mucin accumulation within the right bronchus (B10). (**C**) Histopathological findings of the bronchoscopic biopsy specimen demonstrating mucinous material containing metastatic breast cancer cells. HE, haematoxylin and eosin stain; ER, estrogen receptor; HER2, human epidermal growth factor receptor 2

**Fig. 2:**
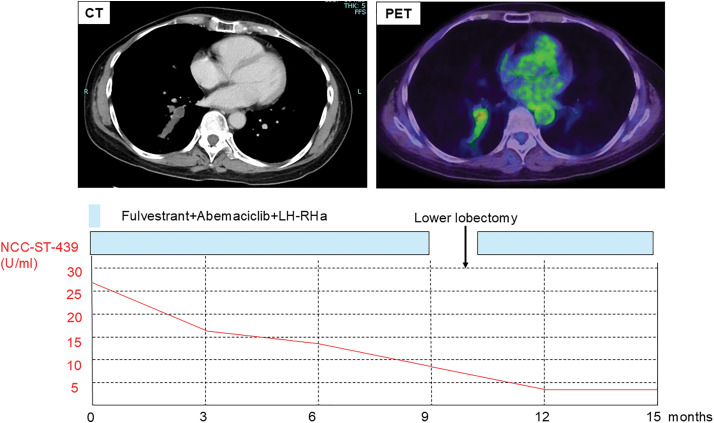
Imaging and tumor marker changes after 9 months of systemic therapy. After treatment with fulvestrant, abemaciclib, and a luteinizing hormone–releasing hormone agonist, serum NCC-ST-439 levels gradually declined, and mild regression of the metastatic lesion was observed without the emergence of new metastatic sites. LH-RH, luteinizing hormone–releasing hormone

Two years and 8 months after surgery, an elevation in serum NCC-ST-439 level was detected (27.9 U/mL). CT revealed mucin accumulation within the right bronchus (B10) (**Fig. 1A**). Bronchoscopic biopsy was performed, and histopathological examination confirmed MBC (ER/PgR/HER2 = 3b/0/0) (**Fig. 1B** and **1C**). The patient was referred to our hospital for further treatment. No additional metastatic lesions were detected on systemic imaging, and the patient was diagnosed with endobronchial oligometastatic breast cancer. Combination therapy with fulvestrant, abemaciclib (a CDK4/6 inhibitor), and an LH-RH agonist was initiated. After 9 months of treatment, serum NCC-ST-439 levels gradually declined, and mild regression of the metastatic lesion was observed without the emergence of new metastatic sites. PET revealed no significant increase in SUV (**Fig. 2**). Germline genetic testing for hereditary breast cancer (including BRCA1/2) was not performed due to patient preference at the time of recurrence. Oligometastatic disease was defined as a solitary metastatic lesion confined to a single organ. Systemic staging with contrast-enhanced CT and PET/CT revealed no additional metastatic lesions, and brain imaging and bone evaluation showed no evidence of disease elsewhere.

**Fig. 3:**
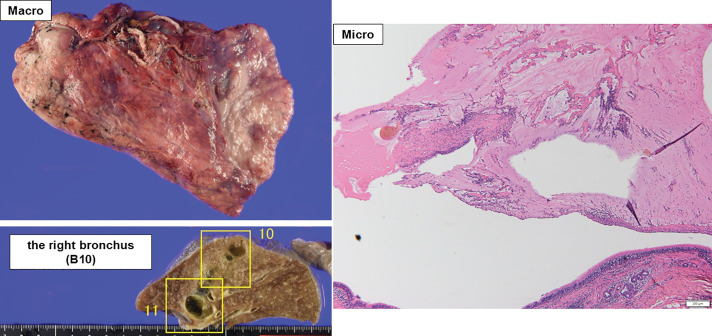
Histopathological findings of the resected specimen. Mucinous material and fibrous tissue were observed at the site corresponding to the presumed metastatic lesion; however, no viable malignant cells were identified, consistent with pathological complete response.

Based on these findings and following a thorough discussion with the patient, robot-assisted right lower lobectomy with ND2a-1 lymph node dissection was performed. Histopathological examination of the resected specimen revealed no viable tumor cells, leading to a diagnosis of pCR (**Fig. 3**). Postoperatively, treatment with fulvestrant, abemaciclib, and an LH-RH agonist was continued; however, abemaciclib was discontinued 3 months after surgery because of severe pruritus. The patient has remained progression-free for one year and 9 months following pulmonary resection. pCR was defined as the absence of viable tumor cells in the resected bronchial lesion and all dissected lymph nodes. The resected specimen showed prominent treatment-related fibrosis and mucinous material centered on the bronchial wall and adjacent peribronchial tissue, with no residual metastatic focus in lung parenchyma or peribronchial lymph nodes and no findings suggestive of secondary bronchial invasion.

## DISCUSSION

EBM can develop through several clinically described development modes, and its endobronchial involvement has been reported in association with particular bronchial sites.^5)^ Clinical symptoms include dyspnea, cough, and hemoptysis; however, many cases remain asymptomatic,^6)^ as observed in the present case. Because diagnosis is established by histological confirmation, bronchoscopic examination is a useful diagnostic modality when EBM is suspected based on imaging findings or clinical presentation.^7)^ Furthermore, when breast cancer is the primary tumor, pathological identification of the metastatic lesion, including assessment of tumor subtype, is crucial for treatment selection. Receptor discordance between primary and metastatic lesions, such as PgR loss in this case, has been reported and supports the importance of re-biopsy for treatment selection.

For HR–positive/HER2–negative MBC, endocrine therapy combined with CDK4/6 inhibitors is the current standard of care and has been shown to improve clinical outcomes; however, CR rates remain low, at approximately 2% in both the PALOMA-2 and MONARCH-3 trials.^8,9)^ Even when radiological CR is achieved, it remains uncertain whether this represents true disease eradication, and surgical resection of metastatic lesions is generally not recommended. Nevertheless, several reports have suggested that resection of oligometastatic disease may be associated with improved prognosis.^10–12)^ In the present case, systemic therapy resulted in favorable disease control, positron emission tomography demonstrated no significant metabolic activity, and no new metastatic lesions were detected. After thorough discussion with the patient regarding the potential benefits and risks, pulmonary resection was therefore performed. Histopathological examination of the resected specimen revealed a pCR, and the patient has subsequently maintained long-term progression-free survival. These findings suggest that, in carefully selected patients with oligometastatic disease, surgical resection following effective systemic therapy may be considered as a treatment option. Non-interventional observation and local therapies (including SBRT or endobronchial interventions) were discussed as alternatives. However, pulmonary resection was selected after multidisciplinary discussion, considering durable local control, prevention of future airway complications (e.g., obstruction or hemoptysis), and the need for pathological confirmation of treatment response, in accordance with the patient’s preference.

In addition, abemaciclib was discontinued for more than 18 months in the present case because of treatment-related pruritus. This decision was made after careful discussion with the patient regarding the potential risk of disease progression, and no evidence of new metastatic disease has been observed during the extended drug-free period. In HER2–positive MBC, several reports have suggested that discontinuation of anti-HER2 therapy after achieving CR may be feasible without subsequent disease progression.^13,14)^ However, the optimal treatment duration and reliable biomarkers for treatment discontinuation remain inadequately defined. In HR–positive/HER2–negative breast cancer, limited data are available regarding patients who achieve pCR following CDK4/6 inhibitor–based therapy. Although such patients may potentially maintain favorable outcomes with endocrine therapy alone, discontinuation of CDK4/6 inhibitors should be approached with caution. Further prospective, multicenter clinical trials are warranted to clarify optimal treatment strategies in this setting.

## CONCLUSIONS

We report a case of endobronchial oligometastatic breast cancer in which pCR was achieved following endocrine therapy combined with a CDK4/6 inhibitor, after which robot-assisted pulmonary resection was performed. The patient has maintained a favorable outcome with postoperative endocrine therapy alone.

## DECLARATIONS

### Availability of data and materials

All data generated or analyzed during this study are included in this published article.
